# Expression of Apoptotic Markers Bcl-2 and Bax in the Vascular Wall

**DOI:** 10.17691/stm2021.13.2.05

**Published:** 2021-01-01

**Authors:** E.A. Klimentova, I.A. Suchkov, A.V. Shchulkin, A.P. Glazkova, R.E. Kalinin

**Affiliations:** Department of Cardiovascular, X-ray Endovascular, Operative Surgery, and Topographic Anatomy, Ryazan State Medical University, 9 Vysokovoltnaya St., Ryazan, 390026, Russia; Professor, Department of Cardiovascular, X-ray Endovascular, Operative Surgery, and Topographic Anatomy, Ryazan State Medical University, 9 Vysokovoltnaya St., Ryazan, 390026, Russia; Associate Professor, Department of Pharmacology and Pharmacy, Faculty of Supplementary Professional Education, Ryazan State Medical University, 9 Vysokovoltnaya St., Ryazan, 390026, Russia; Staff Anesthesiologist, Ryazan Regional Clinical Hospital, 3a Internatsionalnaya St., Ryazan, 390039, Russia; Professor, Head of the Department of Cardiovascular, X-ray Endovascular, Operative Surgery, and Topographic Anatomy, Ryazan State Medical University, 9 Vysokovoltnaya St., Ryazan, 390026, Russia

**Keywords:** atherosclerotic plaque, apoptotic proteins, Bax and Bcl-2 proteins, lower limb arterial atherosclerosis, cholesterol

## Abstract

**The aim of the study** was to assess the levels of Всl-2 and Bax proteins in the vascular wall and their correlation with serum cholesterol in patients with stage III–IV atherosclerosis obliterans of lower limb arteries.

**Materials and Methods:**

The study included 32 patients with stage III–IV atherosclerosis obliterans of the lower limb. Samples of intraoperative material (all three layers of the vascular wall) including an atherosclerotic plaque (AP) were taken during primary open surgery on major leg arteries. As a control, we used samples of the arterial wall without visible signs of atherosclerosis. Based on AP ultrasonography, the patients were divided into two groups: with APs of mixed echogenicity and with hyperechoic (calcified) AP. The vascular samples were crushed and homogenized for further measurements of Всl-2 and Bax proteins; in a separate setup, cholesterol in blood serum was measured.

**Results:**

In patients without atherosclerotic changes, the level of the anti-apoptotic protein Bcl-2 in the arterial wall was 1.25 ng/mg, and that of the pro-apoptotic protein Bax — 4.7 ng/mg. In the case of APs of mixed echogenicity, the expression of Bcl-2 was 1.8 ng/mg (p=0.143) and that of Bax — 5.1 ng/mg (p=0.834), with no significant differences from AP-free vascular wall samples. In the arterial wall containing a heterogeneous calcified AP, the expression of Bcl-2 was 0.9 ng/mg (p=0.143), In contrast, the level of Bax was 6.8 ng/mg, which showed its significant increase as compared with the non-AP controls (p=0.02). In the cases with predominantly hyperechoic AP, the expression of Bcl-2 was significantly lower (p=0.036), and that of Bax — significantly higher (p=0.036) in comparison with AP of mixed echogenicity. In patients with hyperechoic AP, we found a negative correlation between the Bax and Bcl-2 values ( r=–0.315) and a positive correlation between the Bax expression and serum cholesterol (r=0.617).

**Conclusion:**

In arterial walls with hyperechoic (calcified) APs, the expression of anti-apoptotic protein Bcl-2 is reduced, and that of pro-apoptotic protein Bax is increased, which indicates the apoptosis activation in advanced atherosclerotic lesions. In patients with such APs, elevated cholesterol levels directly correlate with the increased expression of pro-apoptotic Bax protein (r=0.617).

## Introduction

Large groups of population in developed countries all over the world suffer from atherosclerosis of coronary, carotid, or peripheral arteries. Development of atherothrombotic complications remains the main cause of lethal outcomes [1–4].

An important role in maintaining the physiological cellular homeostasis is played by the mechanism of apoptosis, i.e. programmed cell death. Dysregulation of apoptosis is associated with infectious and oncological diseases, as well as with atherosclerosis [5]. Among other factors, atherosclerosis is caused by the accumulation of oxidized low-density lipoproteins (oLDL) and inflammatory cytokines in the vascular wall. Apoptosis of macrophages, smooth muscle cells (SMCs), and endothelial cells contributes to arterial atherosclerosis as well [6]. Werner et al. [7] showed that an increase in the number of apoptotic micro-particles led to the development of severe endothelial dysfunction due to increased endothelial permeability, followed by migration of inflammatory cells and proliferation of SMCs. Others [8] found that a decrease in the level of microRNA-34a facilitated the growth of endothelial cells and inhibited cell apoptosis in an atherosclerotic plaque (AP) by activating the anti-apoptotic protein Bcl-2; this observation suggested a promising therapeutic option for atherosclerosis.

It is known that APs contain a large number of dead cells, which can account for up to 80% of the total AP structure. A ruptured fibrous cap in the “necrotic core” of an AP is considered a major cause of arterial thrombosis. Most of the studies on apoptosis of vascular wall cells were carried out in animals. It was shown that in the early stages of atherosclerosis, cell apoptosis counteracted and balanced cell proliferation. In the later stages though, apoptosis of SMCs and macrophages prevailed and led to the development of lipid-enriched necrotic areas [9].

The Bcl-2 protein family is one of the major players in the apoptosis system; these proteins participate in the regulation of the mitochondrial membrane permeability. The proteins of this family are classified as either proapoptotic (Bax, Bak, etc.) or anti-apoptotic (Bcl-2, etc.). The ratio between these two groups is considered predictive of the forthcoming cell death. After receiving a specific signal, Bax or Bak proteins undergo conformational changes and move into the mitochondrial membrane, where they promote the release of cytochrome C into the cytosol [10, 11].

There are studies on Bcl-2 and Bax proteins in coronary and carotid arteries of animals. However, we found no reports on the expression of these proteins in vascular wall samples taken from patients with lower limb atherosclerosis obliterans (ASO). Yet, the status of these apoptotic markers in ASO patients could provide additional information on the pathogenesis and complications of atherosclerosis.

**The aim of the study** was to assess the expression of Всl-2 and Bax proteins in the vascular wall and their correlation with serum cholesterol in patients with stage III–IV atherosclerosis obliterans of lower limb arteries.

## Materials and Methods

The study included 32 male patients (63.4±7.9 years old) with stage III–IV ASO who were treated at the Unit of Vascular Surgery of the Ryazan Regional Clinical Hospital (Russia). Upon admission, all patients underwent routine diagnostic tests including angiography and duplex ultrasonography of lower limb arteries.

After receiving informed consent from the patients, samples of the arterial wall were taken during surgery on main leg arteries. The sampled material represented all three layers of a vascular wall segment containing an AP. As a control (n=12), we used samples of the arterial walls obtained posthumously during organ explantation from subjects without ASO.

Based on the ultrasound characteristics of their APs, patients were divided into two groups: with mixed echogenicity (group 1, n=16) and with hyperechoic (calcified) AP (group 2, n=16) (Table 1).

**Table 1:** Characteristics of the studied samples

Ultrasound of the vascular characterization wall	Stage of the disease		Number of samples	Sampled vessel		
	III	IV		External iliac artery	Femoral artery	Popliteal artery
Intact arterial wall	—	—	12	4	4	4
Atherosclerotic plaque with mixed echogenicity	10	6	16	4	8	4
Hyperechoic (calcified) atherosclerotic plaque	6	10	16	3	7	6

The withdrawn sample was crushed and added with a lysis buffer (Thermo Fisher Scientific, USA). The mixture was centrifuged at 24,000 rpm for 60 s at 2°C. The resulting homogenate was spinned down again at 1000 g for 10 min at 2°C. In the resulting supernatant, the amount of Bcl-2 protein was determined using a commercial ELISA kit (Thermo Fisher Scientific) and the Bax protein — with a Cloud-Clone Corporation kit (China, USA). The results were then recalculated per protein content; the latter was measured by using the Bradford method with Coomassie Plus (Bradford) Assay Kit (Thermo Fisher Scientific).

**Statistical data analysis** was performed using the Statistica 10.0 software package. Since the primary data deviated from the normal distribution pattern (according to the Shapiro–Wilk test, p<0.05), nonparametric tests were used for data analysis. Specifically, the Wilcoxon test was used to compare two interdependent groups, the Mann–Whitney U test — to compare two independent groups, and the Spearman’s test — for correlation analysis. The results were considered statistically significant at p<0.05.

## Results

Our study showed that in the arterial wall without atherosclerotic changes (control group), the level of Всl-2 was 1.25 ng/mg protein, Bax — 4.7 ng/mg, and the Всl-2/Bax ratio was 0.26. The serum cholesterol level was 4.2 mmol/L.

In the arterial wall containing an AP of mixed echogenicity, Bcl-2 was present at 1.8 ng/mg, Bax — 5.1 ng/mg, and the ratio of Bcl-2 to Bax was 0.3. The level of serum cholesterol was 4.4 mmol/L. The above values did not significantly differ from the respective values in the control group.

In the arterial wall containing a heterogeneous (calcified) AP, the level of Bcl-2 was 0.9 ng/mg protein, that of Bax — 6.8 ng/mg, and their ratio was 0.13. The cholesterol level was 7.0 mmol/L, which was significantly higher than that in control (p<0.05). The Bax protein level was significantly (p<0.05) increased in comparison with its level in the arterial wall without atherosclerosis (Table 2).

**Table 2:** Levels of proteins Bcl-2 and Bax in vascular wall homogenates and cholesterol in the blood serum (Me [Q1; Q3])

Segments of the arterial wall	Bcl-2 (ng/mg)	Bax (ng/mg)	Bcl-2/Bax	Cholesterol (mmol/L)
Arterial wall without visible signs of atherosclerosis	1.25 [1.21; 1.30]	4.7 [3.8; 5.6]	0.26 [0.20; 0.30]	4.2 [3.2; 4.3]
Atherosclerotic plaque with mixed echogenicity	1.80 [1.60; 1.83]	5.1 [1.3; 5.1]	0.30 [0.40; 1.30]	4.4 [3.2; 5.7]
Hyperechoic (calcified) atherosclerotic plaque	0.90 [0.60; 1.20]*	6.8 [6.3; 7.1]*	0.13 [0.09; 0.14]*	7.0 [4.2; 7.3]

* Statistically significant differences (p<0.05) from values for atherosclerotic plaque of mixed echogenicity.

In APs with a predominance of the hyperechoic component, the expression of Bcl-2 was significantly lower (p<0.05), and the expression of Bax was higher (p<0.05) in comparison with their expression in APs with mixed echogenicity.

**Figure 1:**
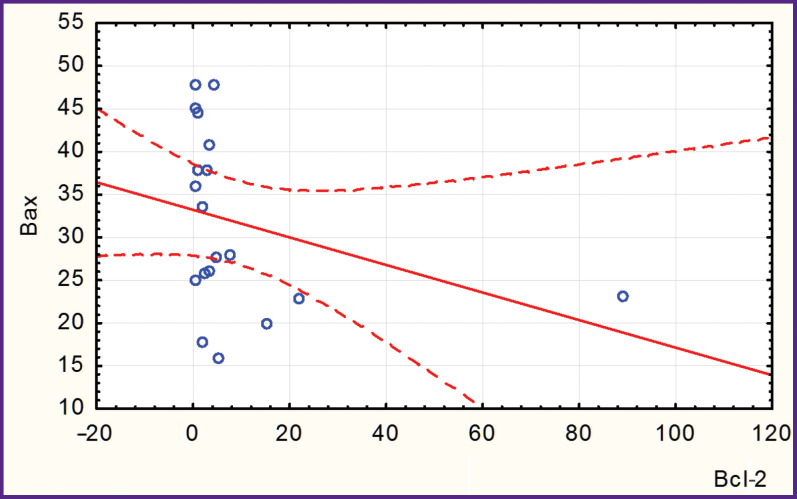
Negative correlation between the Bcl-2 and Bax values in patients with hyperechoic (calcified) atherosclerotic plaque

**Figure 2:**
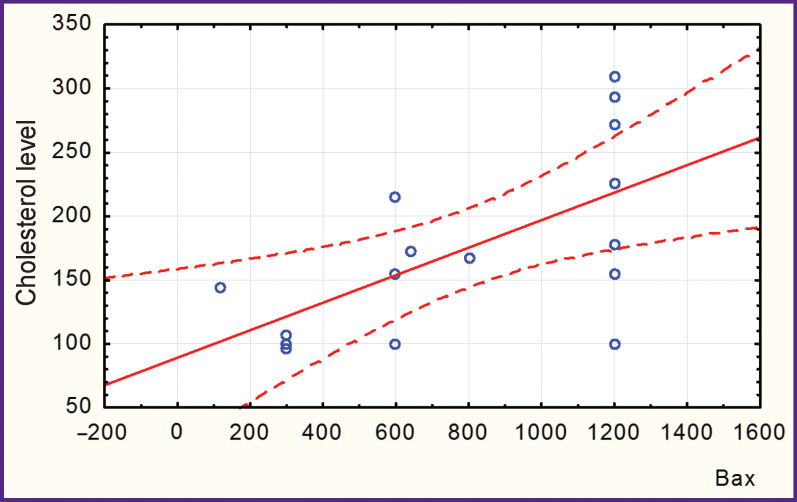
Correlation between Bax values and cholesterol levels in patients with hyperechoic (calcified) atherosclerotic plaque

In patients with hyperechoic (calcified) AP, there was a negative correlation between the levels of Bax and Bcl-2 (r=–0.315; Figure 1) and a positive correlation between the level of Bax and that of serum cholesterol (r=0.617; Figure 2).

## Discussion

In this study, the levels of Bcl-2 and Bax proteins expressed in the intact arterial wall of human peripheral arteries were determined. Normally, cells of an intact vessel demonstrate highly efficient clearance of apoptotic bodies by phagocytes and neighboring cells without the development of an inflammatory reaction, which is necessary for the normal development and functioning of the cell [12].

In atherosclerotic lesions, the expression of these proteins changes in two opposite ways. In hyperechoic (calcified) APs, the level of Bcl-2 is reduced, and the level of Bax is increased as compared with their levels in APs of mixed echogenicity. This may be due to the fact that cell apoptosis inside an AP is associated with vascular wall remodeling, a release of IL-1 and IL-8, an activation of monocyte chemoattractant proteins, and an increased prothrombogenicity following thrombin activation. The data obtained in this study are consistent with the results of other animal studies [13, 14], which showed that in early atherosclerotic lesions, the number of cells undergoing apoptosis did not exceed 10% but this number increased as the disease progressed. The proapoptotic markers Fas and caspase-3 were detected in the endothelium and the SMC media component of the arterial wall close to the lumen of the vessel. The antiapoptotic protein Bcl-2 was found in macrophages and SMCs in the deeper layers of the vascular wall.

The present study shows that the increased level of Bax is associated with the deposition of calcium in the plaques. This is due to the fact that the matrix vesicle-like structures in the plaques are considered to be apoptotic residues that should be instantly cleared by adjacent phagocytes. In the predominantly cell-free lipid core of the AP, phagocytosis of apoptotic cells can be slowed by oLDL that compete with these cells for binding to phagocytes. The remaining non-phagocytosed apoptotic cells can undergo secondary necrosis, which is subsequently calcified.

It should be noted that the apoptotic bodies themselves can accumulate and bind calcium. In the literature, we have found only one report on this subject. Thus, in an *in vitro* experiment, Proudfoot et al. [15] showed that a caspase inhibitor reduced the calcification of cells in APs.

In this study, the observed correlation between the levels of Bax and serum cholesterol suggests that an increased amount of cholesterol may activate the mitochondrial apoptosis pathway. Cholesterol is part of the cell membrane and, when increased, can change its permeability, contributing to the destabilization and rupture of lysosomes, which, in turn, causes cell death. In addition, the proapoptotic effect of cholesterol may be due to a decrease in antioxidant enzymes (superoxide dismutase and glutathione peroxidase) with an increased generation of reactive oxygen species. According to one study [16], oxidized LDL are able to stimulate receptor-mediated apoptosis followed by activation of caspases and pro-apoptotic protein p53. Our study suggests that the elevated serum cholesterol in patients with lower limb ASO is paralleled with elevated markers of the mitochondrial apoptotic pathway, namely Bcl-2 protein.

To improve the accuracy and reliability of predicting the progression of atherosclerotic lesions in the vascular wall and to facilitate the search for novel therapies, further studies on apoptotic proteins and their relationship with serum cholesterol are warranted.

## Conclusion

The expression of anti-apoptotic protein Bcl-2 is reduced, and that of proapoptotic protein Bax increased in hyperechoic (calcified) atherosclerotic plaques, which indicates the activation of apoptosis in advanced atherosclerotic lesions.

An increased amount of cholesterol in the blood serum of patients with lower limb atherosclerosis obliterans is associated with the activation of the mitochondrial apoptotic pathway in calcified atherosclerotic plaques.
